# Chicken genome analysis reveals novel genes encoding biotin-binding proteins related to avidin family

**DOI:** 10.1186/1471-2164-6-41

**Published:** 2005-03-18

**Authors:** Einari A Niskanen, Vesa P Hytönen, Alessandro Grapputo, Henri R Nordlund, Markku S Kulomaa, Olli H Laitinen

**Affiliations:** 1 NanoScience Center, Department of Biological and Environmental Science, FIN-40014 University of Jyväskylä, Finland; 2 A.I. Virtanen Institute, Department of Molecular Medicine and Biotechnology, University of Kuopio, P.O. Box 1627, FIN-70120 Kuopio, Finland; 3 Present address: Institute of Medical Technology, FIN-33014 University of Tampere, Finland

## Abstract

**Background:**

A chicken egg contains several biotin-binding proteins (BBPs), whose complete DNA and amino acid sequences are not known. In order to identify and characterise these genes and proteins we studied chicken cDNAs and genes available in the NCBI database and chicken genome database using the reported N-terminal amino acid sequences of chicken egg-yolk BBPs as search strings.

**Results:**

Two separate hits showing significant homology for these N-terminal sequences were discovered. For one of these hits, the chromosomal location in the immediate proximity of the avidin gene family was found. Both of these hits encode proteins having high sequence similarity with avidin suggesting that chicken BBPs are paralogous to avidin family. In particular, almost all residues corresponding to biotin binding in avidin are conserved in these putative BBP proteins. One of the found DNA sequences, however, seems to encode a carboxy-terminal extension not present in avidin.

**Conclusion:**

We describe here the predicted properties of the putative BBP genes and proteins. Our present observations link BBP genes together with avidin gene family and shed more light on the genetic arrangement and variability of this family. In addition, comparative modelling revealed the potential structural elements important for the functional and structural properties of the putative BBP proteins.

## Background

Chickens are known to produce several different proteins which bind biotin in a non-covalent fashion. One of them is avidin, which is expressed by oviduct cells upon progesterone induction and is then transferred to the egg-white where it constitutes a minor fraction of the total protein content of the egg-white [1]. Independently of progesterone, avidin expression is also induced by an inflammation response in almost all of the studied chicken tissues [2]. Another biotin-binder, called literally biotin-binding protein (BBP), is presumably induced by estrogen [3] and secreted from the liver into chicken plasma [4]. From plasma, the BBP is thought to be deposited in egg-yolk [5]. In addition, Seshagiri and Adiga found another egg-white BBP, distinct from avidin, the biochemical characteristics of which resemble those reported for yolk BBP [6].

The affinities that avidin and yolk BBP exhibit toward biotin are extremely high, the dissociation constant being femtomolar for avidin [1] and picomolar for BBP [7]. According to the published data, the yolk BBP serves as a biotin reserve for the developing embryo and hence it is saturated with the vitamin [5]. In contrast, avidin in egg-white is mainly found as an apoprotein and it is assumed to function as an antimicrobial agent that harvests free biotin from its environment [1,2]. Because of its high affinity to biotin, avidin has long been used as a separation, labelling and targeting tool in various bioscience fields [8].

The yolk BBP has been further characterised to consist of two different forms, BBP-I and BBP-II [3,9]. It has been proposed that BBP-I is the primary gene product (67 kDa) that is converted by proteolytic cleavage to BBP-II (19 kDa) [4]. The biological function of BBP-I is believed to be a general biotin transporter in plasma, whereas the actual deposition role in egg-yolk is reserved for BBP-II [3]. BBP-II is a tetrameric protein, like avidin, and is composed of subunits homologous to each other. BBP-I has been thought to be a pseudotetramer containing four binding domains in a polypeptide chain and its gene should, therefore, contain four subsequent repeats encoding for similar peptide sequences [4,9]. The egg-white BBP was also reported to exist in a large form similar to yolk BBP-I [6]. Interestingly, some egg-laying species, such as turkeys and alligators, showed only one type of BBP in the yolk of their eggs [10].

Despite their similar function, some biochemical properties of BBPs and avidin are different. The pI of the chicken yolk BBP (not defined which form) was reported to be 4.6 [7] in contrast to avidin which has a highly basic pI (≈ 10.4) [1]. BBP-I exhibits higher thermostability, being active at 60°C, whereas BBP-II is denatured at temperatures above 40°C [10]. Bush and White III have published the N-terminal amino acid sequences for both chicken yolk BBP forms, which are highly similar to each other and also resemble the avidin N-terminal sequence [4,10]. BBP-I has been proposed to be a glycoprotein [7] whereas BBP-II is shown to be nonglycosylated [10]. Avidin is known to contain one N-linked carbohydrate moiety per subunit [1]. Differences in the radiobiotin exchange rates between these two BBP forms have also been observed: BBP-I showed slower exchange than BBP-II [10].

The published N-terminal sequences, the similar overall sizes of the proteins and the tetrameric appearance of BBP-II as well as the reported high biotin-binding affinities suggest that the BBPs could be related to avidin. Structurally and functionally, avidin is considered to be a member of the larger protein superfamily called calycins [11]. These proteins form a large and divergent family of relatively small extracellular proteins which typically bind small hydrophobic ligands. Two particular groups of calycin protein family, the lipocalins and avidins, are β-barrels composed of eight antiparallel β-strands. The ligand is bound inside the protein at one end of the β-barrel [12]. An interesting feature among the lipocalins and avidins is a structural signature wherein a conserved basic amino acid residue, close to the last β-strand, packs over a specific tryptophan residue on the first β-strand and forms hydrogen bonds with the short 3_10 _helix prior to the first β-strand [11].

Because the genes, genomic locations, cDNAs or full amino acid sequences of chicken BBPs are not known, it is impossible to evaluate their true relationship to avidin or, in a broader sense, to the calycin protein superfamily. New hope to solve this enigma aroused when the first draft of the chicken genome was published in March 2004 [13,14]. In addition to the genome project, a comprehensive collection of chicken cDNAs is also in progress [15].

In the current study we searched these databases in order to find the cDNAs and genes for BBPs. Indeed, we found two independent cDNAs whose translated amino acid sequences fitted well to the published N-terminal sequences of BBPs. The genomic fragments corresponding to these cDNAs were also identified and analysed. They showed features similar to those of the avidin gene family members. Interestingly, one of these BBP gene candidates is located together with the avidin gene family in the chicken chromosome Z [16]. In addition, more evidence supporting the previous hypothesis of the high recombination frequency in the avidin gene family is gathered. One of the two putative BBPs was found to significantly resemble avidin, showing a theoretical molecular mass and pI close to those of avidin, whereas the other showed theoretical characteristics fitting more closely to those published for BBPs. Almost all amino acids important for biotin binding in avidin [17] are conserved in both of these supposed BBPs. Neither of the found cDNAs/genes, however, encodes a protein composed of four similar domains as expected for the isolated pseudotetrameric BBP-I [3]. Instead, the encoded proteins show calculated molecular masses corresponding to one BBP domain per polypeptide. *In silico *analysis of these genes as well as modelled structures of the putative BBP proteins are presented.

## Results

### Database queries and sequence analyses

The database searches revealed three cDNA hits which showed significant similarity for the used N-terminal query sequences. The public accession codes for these hits are [GenBank:BX930135], [GenBank:BX932076] and [GenBank:BX936151]. However, detailed analyses suggest that one of the hit sequences, [GenBank:BX932076], is identical to sequence [GenBank:BX936151] but differentially spliced (first intron being present). The reason for that might be a non-mature cDNA clone obtained during the cDNA library preparation [15]. The two other hits, [GenBank:BX930135] and [GenBank:BX936151] were therefore chosen for further analysis (Table 1). For clarity these two sequences are called *BBP-A *[GenBank:BX930135] and *BBP-B *[GenBank:BX936151].

**Table 1:** The BBP cDNA sequences found from the database and theoretical biochemical characters of the putative BBP proteins. Numbering of the sequences according to avidin sequence.

	BBP-A	BBP-B	Method
N-glyc (rank)	^17^NMTI^20 ^(9/9)	^74^NATT^77 ^(5/9)	NetNglyc^b^
Molecular mass (Da)^a^	13845.8	16404.5	ProtParam^c^
pI^a^	9.75	5.88	ProtParam
Number of residues^a^	124	148	ProtParam
Extinction coeff. (280 nm)^a^	24160	35660	ProtParam

^a^The signal sequence is cut at the most probable cleavage site according to prediction and published N-terminal sequences [4] (BBP-A: 1/2 AS-RKCE; BBP-B: -2/-1 TP-VERK).

^b^[54]

^c^[53]

**Figure 1:**
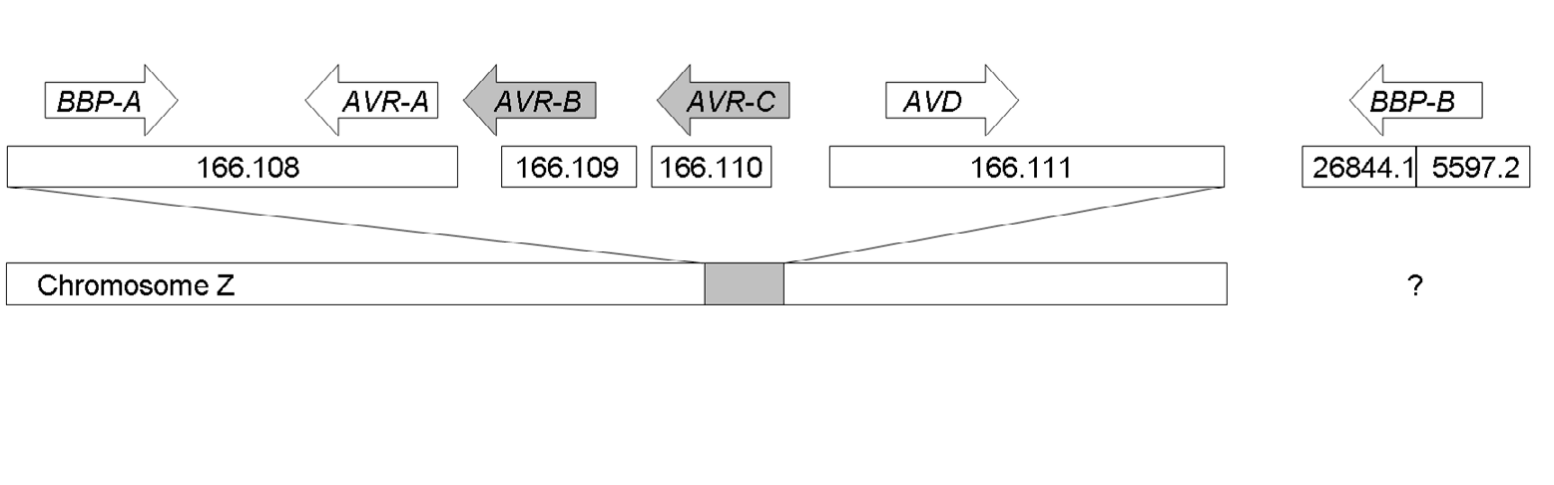
**Schematic presentation of the genomic locations and orientations of the genes. **The genomic location of the *BBP-B *gene is unknown, whereas contigs 166.108-111 reside in the Z-chromosome as schematically shown in lower part of the picture. According to previous studies, the most probable location is in the q21 telomeric region of the Z-chromosome (Alhroth *et al*., 2000).

**Figure 4:**
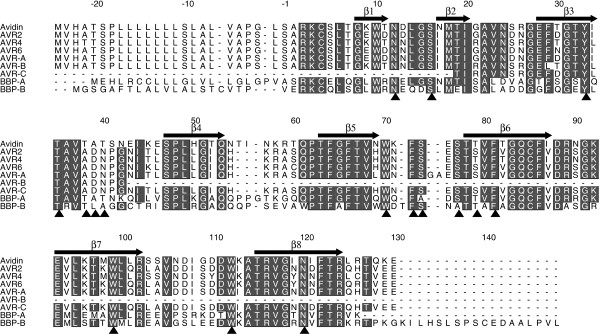
**Sequence alignment. **The sequence alignment of avidin, avidin related proteins AVR2, AVR4 and AVR6 (Laitinen *et al*., 2002), translated AVR genes *AVR-A*, *AVR-B *and *AVR-C *from chicken genome database and putative biotin-binding proteins BBP-A and BBP-B. Numbering and secondary structures are according to avidin sequence. Completely conserved amino acids are shown in white with black background. Residues with direct contact to biotin in avidin (Livnah *et al*., 1993) are indicated with black triangles.

The gene containing the fully identical sequence to *BBP-A *cDNA with three introns was found in chicken genome database in Contig166.108. Two contig-sequences (Contig55972.2 and Contig26844.1) containing parts of *BBP-B *were found in the database. These were manually joined together (Figure 1). The final product contained five changes in the nucleotide sequence when compared to *BBP-B *cDNA ([GenBank:BX936151]), causing differences in three amino acid residues close to the C-terminal part of the protein (N118I, V119L, F120L). The avidin gene and three avidinrelated genes were also found from the chicken genome database. The gene of *BBP-A *and a novel allele of one of the previously cloned *AVR*s (or a novel avidin-related gene), which we named *AVR-A *were found in the same Contig166.108. *AVR-A *was similar to *AVR*2 and *AVR*6 (Figure 4) [16]. *BBP-A and AVR-A *genes point towards each other (*BBP-A*→ ←*AVR-A*) separated by an intergenic distance of 8.1 kB. A chicken repeat 1 (CR1) element [18] was found between these two genes. The distance between *AVR-A *and CR1 was 0.6 kB while the distance between the *BBP-A *gene and CR1 element was 6.1 kB. CR1 pointed towards *BBP-A *and it was in parallel orientation with *AVR-A*. Previously, Wallén *et al*. have reported CR1 elements located 1.4–2.1 kB upstream from the 5'-ends of *AVR4 *and *AVR5 *genes and pointing towards the genes [19]. Contig166.109 contains a partial gene (named *AVR-B*) clearly resembling *AVR4*. However, it has a mutation that converts Phe-29 in the AVR4 protein to leucine. In addition, Contig166.110 contains a partial gene (named *AVR-C*) resembling *AVR2 *with the exception that it codes for Ser and Arg in positions 25 and 26 (as in avidin) instead of Asp and Asn found in AVR2 (Figure 4). Finally, Contig166.111 contains the avidin gene (Figure 1).

**Figure 2:**
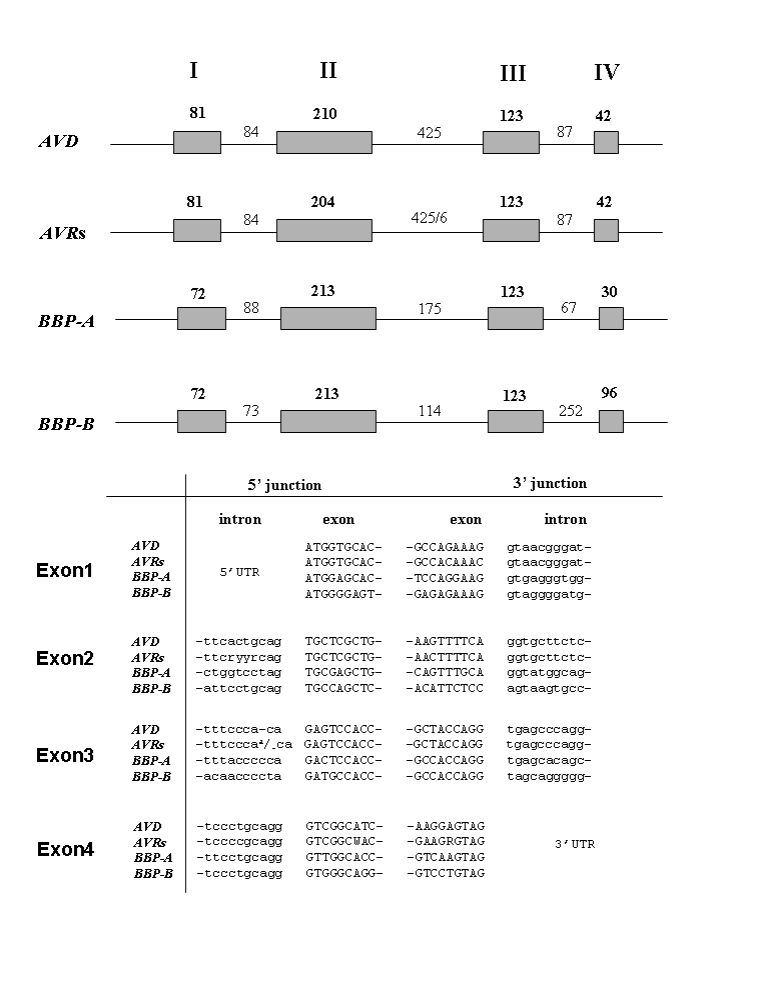
**Structural organization of the avidin and biotin-binding protein genes. **The coding region of each gene is composed of four exons and three introns (upper panel). The respective exon and intron sizes (in nt) are indicated. Sequences at the exon-intron junctions are presented in the lower part of the figure. *AVR *sequences are consensus sequences of different alleles/genes reported in this study. ^a^/- is a variable site among *AVR*s meaning either a gap or adenine.

Alignment of *BBP *cDNAs with their corresponding DNA contig sequences revealed that both of these genes contain four exons and three introns, as shown for avidin and avidin-related genes [20]. The exon and intron lengths of the *BBP *genes and their comparison with the avidin gene structure are shown in Figure 2. The fourth exons are cut after the stop-codon, and the first exons (N-terminus) are cleaved before the ATG starting open reading frame. The sizes of the exons are relatively similar with the exception of the fourth exon of *BBP-B *which encodes 96 amino acids residues, compared to the 30–42 residues in *BBP-A *and *AVD/AVRs*, respectively. The number of variable sites among the exons ranged from 25% (fourth exons) to 58% (second exons). The first intron is similar in size in all compared sequences, whereas the second intron is considerably longer in the avidin and *AVR *genes (about 425 bp) than in the *BBP *genes (175 bp in *BBP-A *and 114 bp in *BBP-B*). On the contrary, the third intron is longer in the *BBP-B *gene (252 bp) than in the avidin/*AVR *genes (87 bp) (Figure 2). The number of variable sites among intron sequences ranged from 24% in the third intron to 59% in the first intron.

A high similarity among the genes was observed at the exon/intron junctions as shown in Figure 2. Sequence divergence (p-distance) among avidin and *BBP *genes ranged from 1.4% between *AVR4 *and *AVR-B *and 48.5% between *AVR-B *and *BBP-B *(Table 2). Similar values were obtained when sequence divergence among exons only or introns only (the combined sequence) were analysed (not shown).

**Table 2:** Pairwise p-distance (below diagonal) and S.E. (above diagonal) between avidin, *AVRs *and BBP genes obtained using MEGA v.3 [40].

	*AVD*	*AVR2*	*AVR4*	*AVR6*	*AVR-A*	*AVR-B*	*AVR-C*	*BBP-A*	*BBP-B*
	
*AVD*		0.009	0.007	0.008	0.009	0.016	0.010	0.018	0.019
*AVR2*	0.083		0.007	0.005	0.004	0.015	0.004	0.018	0.019
*AVR4*	0.054	0.050		0.006	0.007	0.007	0.007	0.018	0.019
*AVR6*	0.085	0.025	0.052		0.005	0.016	0.006	0.018	0.019
*AVR-A*	0.093	0.016	0.055	0.024		0.016	0.005	0.018	0.019
*AVR-B*	0.080	0.070	0.014	0.084	0.077		0.026	0.030	0.031
*AVR-C*	0.095	0.014	0.047	0.032	0.018	0.065		0.021	0.021
*BBP-A*	0.412	0.423	0.434	0.426	0.427	0.434	0.431		0.019
*BBP-B*	0.443	0.440	0.453	0.436	0.441	0.485	0.445	0.452	

**Figure 3:**
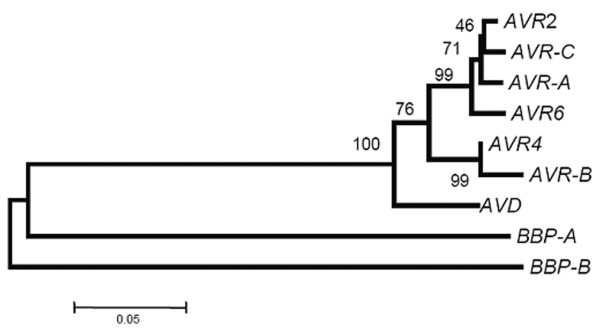
**Evolutionary relationships of genes. **Neighbour joining tree obtained from the gene sequences of avidin, avidin related genes 2, 4 and 6 (*AVR *genes are from Ahlroth *et al*., 2000) and *BBP-A *and *B *genes. The tree was obtained from a pairwise p-distance matrix between sequences as implemented in MEGA v.3 (Kumar *et al*., 2004). Numbers indicate node bootstrap supports.

The phylogenetic relationship of the *AVD*, *AVRs *and biotin-binding protein genes is shown graphically in Figure 3 (the same relationships were obtained from the amino acid sequences; not shown). In the unrooted tree, avidin and avidin related genes formed a well supported cluster, which was the sister group of *BBP-A*. Finally, basal to the tree, was *BBP-B*.

All characterised genes contained a potential promoter region upstream of the coding region according to prediction program used. In the case of *AVR-C*, the upstream region of the gene was not analysed due to a missing sequence. The promoters of *BBP-A *and *BBP-B *contained a TATA sequence TATAAA at position (-30)-(-25) nt upstream of the predicted transcription initiation site. In the case of the *AVD *and *AVR-A/B *genes, the sequence AATAAAA was detected (-31)-(-25) nt upstream of the predicted transcription initiation site. The putative promoter regions contained possible binding sites for several transcription factors (not shown).

### Amino acid primary sequence characteristics

The most obvious difference between the BBP-B, when compared to avidin and BBP-A, is a C-terminal extension which makes it 18 residues longer than avidin and 22 residues longer than BBP-A. The sequence identity between BBP-A and BBP-B is 49%. The identity between the aligned regions of chicken avidin and BBP-A is 59% and between BBP-B and avidin is 47%. The residues involved in biotin binding in avidin [17] are almost perfectly conserved in both the BBP-forms (Figure 4). The only substitutions within these residues are Ser-73 which is replaced with alanine in BBP-A and Ser-75 which is replaced with alanine in BBP-B. Moreover, the T-A-T sequence in avidin (residues 38–40), that forms bonds with the carboxylic tail of biotin, is conserved in BBP-A, but replaced with T-L-A in BBP-B.

The SignalP signal prediction program suggested the presence of signal peptide in BBP-A at residues (-21)-(-1) (numbering according to sequence alignment, Figure 4) and a cleavage site at position 1/2. Similarly, a signal peptide composed of residues (-21)-(-2) and a cleavage site in position 2/3 [21] or -2/-1 (hidden Markov model [22]) was predicted for BBP-B. It seems, therefore, that these putative BBPs are secreted proteins like avidin [1].

Both BBPs have one possible N-glycosylation site being ^17^Asn-Met-Thr-Ile^20 ^for BBP-A (identical to avidin) and ^74^Asn-Ala-Thr-Thr^77 ^for BBP-B. The prediction shows, however, a low probability for glycosylation to occur in BBP-B.

The cysteine residues (Cys-4 and Cys-83) which form the intrasubunit disulphide bridge in avidin are conserved in both BBPs. In addition, two cysteine residues are found in the putative signal sequence of BBP-A and one in the signal sequence of BBP-B. Furthermore, there are two additional cysteines in BBP-B, one in the position corresponding to Glu-43 in avidin and one located in the middle of its C-terminal extension.

The aromatic amino acids are conserved throughout the sequences. The only exceptions are the two tryptophans found only in BBP-B in the region corresponding to β-sheet 5 in avidin.

### Secondary and tertiary structure characteristics based on the homology modelling

The residues in BBP proteins corresponding to the β-sheet secondary structure elements of avidin are significantly more conserved when compared to the loop regions of avidin.

**Figure 5:**
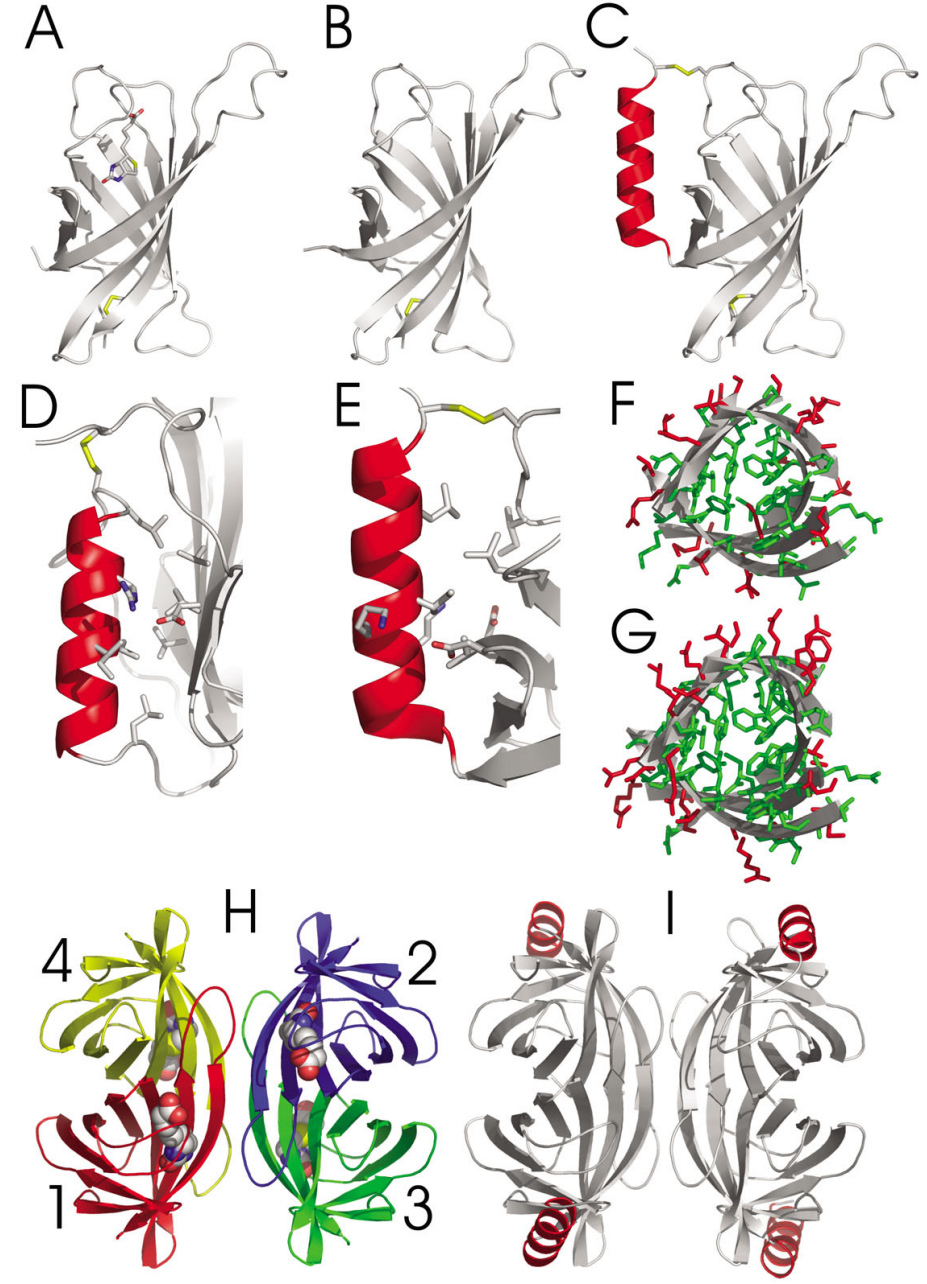
**Comparative modelling of BBPs. **A) Three-dimensional structure of the avidin subunit with bound ligand determined by X-ray crystallography (Livnah *et al*., 1993). B) The modelled structure of the BBP-A subunit and C) BBP-B subunit. Secondary structures are shown as cartoons: α-helix is red and β-sheets are grey. Cysteine bridges are shown as yellow sticks D) View of helix-β-barrel contact in RBP structure determined by X-ray crystallography (Cowan *et al*., 1990). The side-chains forming the contacts are shown as sticks. In E) is an enlargement of the modelled BBP-B helix-β-barrel contact. The conservation of the inner part and changes in the outer part of the modelled barrels of BBP-A (F) and BBP-B (G) are shown when compared to avidin. Conserved side-chains are shown in green and non-conserved are shown in red. The loop regions are omitted from the figure as well as the C-terminal predicted α-helix of BBP-B. H) Avidin tetramer with bound biotins, subunits are numbered according to Livnah *et al*., 1993. I) Tetrameric model of BBP-B. Proposed α-helices are oriented outwards from the possible tetramer.

Overall, the homology modelling strongly suggests avidin-like secondary (Figure 4) and tertiary (Figure 5) structures for both BBPs.

Based on the modelled structures, both BBPs have the common lipocalin-motif: Gly-Xaa-Trp residues close to the N-terminus (residues 8–10 in alignment) and arginine in the last β-strand. This structural signature indicates that BBPs belong to the calycin superfamily together with avidin and streptavidin (which is a bacterial analogue of chicken avidin) [12].

At the tertiary structure level the most striking feature of the BBPs, when compared to avidin, is the conservation of the amino acid residues forming the inner part of the β-barrel. These amino acids also include almost all biotin-binding contact residues (Figures 5F, 5G). The hydrogen bond between biotin and Asp-128 in streptavidin [23], and biotin and the analogous residue Asn-118 in avidin (Hytönen VP *et al., *unpublished results), are known to be important for their ligand binding. The bonding network including this residue comprises bonds between Gln-24 and Asp-128 in streptavidin [24] and Asn-118 and Asp-13 in avidin [17]. The residue corresponding to Asn-118 in avidin is conserved in both BBPs.

The role of the C-terminal extension of BBP-B was hypothesised by modelling. Since there is an orphan cysteine residue near the end of β-strand 4 in BBP-B and another cysteine residue close the end of the C-terminal extension, one could assume a disulphide bridge between these cysteines. Several details support this possibility. Firstly, the region close to the cysteine residue in β-strand 4 in BBP-B seems to be rather hydrophobic (K9L, N17L, T34L, E46I), in comparison with the corresponding region in avidin. This might indicate a presence of a shielding structure in this region (i.e. contact to another protein or peptide) (Figure 5C). Secondly, the distance between the end of β-strand 8 and the cysteine in β-strand 4 is in good agreement with the length of the polypeptide sequence. Thirdly, similar structures are found in structurally similar lipocalin family proteins. For example, retinol-binding protein (PDB code: 1RBP) has a similar α-helix connected by a disulphide bridge in corresponding region [25].

### Quaternary structure: interface-regions

All residues forming the 1–2 interface (numbering according to Livnah *et al. *[17]) in avidin are conserved in all of the studied proteins (Table 3). The 1–4 interface, being the most extensive, shows interesting similarities and differences when compared to that of avidin. Residues Gln-53, Thr-67, Trp-70, Gln-82, Val-103 and Thr-113 in this interface are conserved in all of the studied proteins. Asn-54 in the 1–4 interface has been shown to have a central role in the structurally important hydrogen-bonding network in the avidin structure [17,26]. Interestingly, histidine is found in this position in the AVR-proteins, which are known to be stable tetramers [27,28]. Glutamine in this position in the BBP model-structures seems to be able to form similar contacts between the subunits over the 1–4 interface. Taken together, only 10 out of 21 interacting residues in the 1–4 interface are conserved in BBP-A when compared to avidin, with the value being 8 out of 21 in BBP-B.

**Table 3:** Comparison of interface residues of avidin, putative BBPs and AVR-proteins. Residues at subunit interfaces in avidin are determined according to Livnah *et al*. [17]. Equivalent residues in the other proteins are shown based on their alignment.

Secondary structure and type of interaction of amino acid residues of avidin in different subunit interfaces			Residue in avidin	Differences found in other proteins		
Secondary	M-chain	S-chain		BBP-A	BBP-B	AVRs^a^

**1–4 interface**						

β4	H-bonds		H50	K^b^	R^c^	L
β4	H-bond		Q53	-	-	-
β4		H-bonds	N54	Q^d^	Q^d^	H
B4	H-bond	H-bond	T55	P^b^	Q^b^	gap
L4		H-bond	N57	T^d^	S^d^	K
L4		H-bonds	R59	G^b^	V^b^	A
β5	H-bond		G65	-	A^d^	-
β5	H-bond	H-bond	T67	-	-	-
β5		H-bonds	N69	Q^e^	W^f^	L/H
L5	H-bonds		W70	-	-	-
L5		H-bond	K71	Q^d^	D^b^	N
L5	H-bonds		S73	A^d^	-	-
β6		H-phobic	V78	-	A^d^	-
β6	H-bond		T80	V^f^	A^f^	V
β6		H-bonds	Q82	-	-	-
β7	H-bond		M96	A^b^	T^b^	K
β7		H-phobic	L98	-	M^d^	-
β7		H-bond	R100	-	-	-
β7	H-bond		S101	E^g^	E^g^	L
L7	H-bond		V103	-	-	-
β8		H-bonds	T113	-	-	-

**1–2 interface**						

L7		H-phobic	W110	-	-	-
B8	H-bond		T113	-	-	-
B8	H-bond		V115	-	-	-

**1–3 interface**						

B7	H-bond	H-phobic	M96	A	T^b^	K
B8		H-phobic	V115	-	-	-
B8		H-phobic	I117	T	R^h^	N/Y

^a^Differences found in all AVR-proteins 1–7 in previous studies [27, 31] and current study (AVR-A, B, C)

^b^No interaction according to the model

^c^Salt bridge to D26 in subunit 4 according to the model

^d^Interaction similar to avidin according to the model

^e^Interaction similar to avidin without linking water molecule according to the model

^f^Hydrophobic interaction according to the model

^g^Side-chain hydrogen bond according to the model

^h^Salt bridge to E13 in subunit 3 according to the model

In the 1–3 interface, Val-115 is conserved, whereas both Met-96 and Ile-117 show variance both in BBPs and AVRs. The position of Met-96 shows interesting substitutions in other proteins since this residue faces the identical residue from the neighbouring subunit in avidin structure. According to previous mutagenesis studies this residue is known to be important for the tetrameric quaternary structure of avidin [26,29]. According to the model structure, Arg-117 in BBP-B might form an interesting intersubunit salt bridge with Glu-13 from subunit 3.

## Discussion

The circumstantial evidence has indicated that chicken yolk BBPs may be structurally related to avidin and other members of the avidin family. Therefore we were eager to scan the chicken genome data to evaluate the correctness of this hypothesis. The database queries revealed two independent hits showing high similarity to the published N-terminal sequences of yolk BBPs I and II [4] and, indeed, a potential kinship between BBPs and avidin gene family members was revealed.

The avidin gene belongs to the gene family that has several other members called *AVRs *(avidin related genes). Previously, seven different *AVR*-genes have been cloned [16,20] and the chromosomal location of this gene family has been tracked down to a relatively short region in the telomeric region q21 of the chicken sex chromosome Z [16]. It seems that the number of *AVR *genes varies between individual chickens and even between cells within the same chicken [30]. The deposited genome data, analysed in the present study, support this observation demonstrating a novel assembly of 3 *AVR *genes together in the same cluster with the avidin gene. Interestingly, the two *AVR *genes found in the chicken genome database seem to be novel variants of the formerly cloned *AVRs*, which also support the previous hypothesis of the high recombination frequency within the avidin gene family [30,31].

Our observations link the BBPs to the avidin family for the first time, at the cDNA and gene level. There are many independent features indicating this. Firstly, the found cDNAs encode proteins that are evidently homologous to avidin. Secondly, the genomic location of the *BBP-A *gene close to the avidin gene family supports their relationship. Finally, the exon/intron structures of the *BBP *genes and avidin family genes are similar to each other.

According to the phylogenetic relationships and genome locations of the genes, one scenario for the *BBP/*avidin evolution is as follow: an initial duplication may have occurred leading to the origin of *BBP-B *and the precursor of the *BBP-A/*avidin family, followed by a further duplication leading to the origin *of BBP-A *and the precursor of the avidin family. This could have been followed by the formation of *AVD *and an *AVR *gene and finally the duplication of the latter in several avidin-related genes.

According to molecular modelling, BBP-A and B proteins both showed features that make them suitable for biotin binding. The biotin-binding contact amino acids of avidin [17] were almost perfectly conserved in both BBP sequences. In addition, good conservation of the inner part of the β-barrel in both BBPs is also important for the function of the ligand binding cavity. If we assume that one or both of these putative BBPs represent yolk BBPs I and/or II, other sequence differences should explain their observed weaker dissociation constant for biotin [7]. Similar observation has been done for AVRs which have only a few differences in their biotin-binding residues when compared to avidin, but still exhibit remarkable differences in their biotin-binding affinities [27].

It is probable that both BBP-A and BBP-B form similar tetrameric quaternary structures as avidin. The basis for this assumption is the fact that the conservation of the presumed interface residue patterns were highly similar to those of AVRs, which are also known to form extremely stable tetramers [27,28]. The 1–2 interface, in particular, in which mutations have previously been shown to be extremely important to the stability properties of both avidin and streptavidin [32-34], was perfectly conserved in both BBPs. Hence the existing changes were concentrated on the 1–3 and 1–4 interfaces which are known to tolerate substitutions in avidin and AVRs [27-29]. The yolk BBPs have, however, been reported to be clearly less heat-stable than avidin [10] and, therefore either the sequence differences in the interfaces may explain this difference or the tertiary structure of the BBP barrel may be weaker than that of avidin. Overall, interfaces in the BBP models suggested tetramer formation, since the putative interface regions were hydrophobic. Furthermore, differences at the subunit interfaces of the BBP models, when compared to those of avidin, were at least partially complementary. For example the Thr-80-Val mutation at the 1–4 interface of BBP-A seemed to be in a highly hydrophobic environment.

The most striking feature that distinguished BBP-B from avidin and BBP-A was its extraneous, approximately 20 amino acid residue-long, C-terminal extension. According to modelling, this stretch could form an α-helix and the cysteine residue at the end of this stretch could form a disulphide bridge with another cysteine residue in β-strand 4 (Figure 5C). It is, however, hard to interpret the relevance of this predicted α-helix and the possible effect of the helix and the cysteine bridge to the structural and functional properties of BBP-B. One effect could be that it strengthens the structure of the protein. Interestingly, many members of the lipocalin protein family have similar C-terminal α-helical domains [11,12]. Nonetheless, the exon/intron structure of the avidin gene family is different when compared to the lipocalin family [35,36]. This suggests that even if the overall tertiary structures of these proteins are similar, the evolutionary distance between these lineages is overwhelmingly long, or alternatively these protein families have been developed independently. The manner in which BBP-B has acquired its C-terminal extension, found in lipocalins, remains therefore an enigma. Alternative models for this extension can be done as well; the distance between the beginning of C-terminal extension (Lys-123) of the subunit 1 and the free cysteine in loop 3 of the subunits 3 and subunit 4 is around 40 A (not shown). This is also approximately the length of the fully extended C-terminal extension (Lys-123-Cys-138). Based on these distances both 1–3 and 1–4 intersubunit disulphide-bridges are possible.

In the light of the current study, the story of the chicken BBP proteins gets blurred. As before, we have two possible candidates. However, these cDNAs/genes are able to encode proteins having molecular mass of 14–16.5 kDa, which correspond to only one BBP subunit instead of the four subsequent repeats hypothesized earlier [3,9]. This means that either the database still lacks the genuine yolk-BBP gene or that some other phenomenon, like the abovementioned hypothetical disulphide bridges, must explain the previously reported molecular weight properties of BBP-I. Nonetheless, the fact that cDNAs for BBP-A and BBP-B were isolated from the chicken liver library [15], and that the putative proteins they encode have signal sequences, suggest that these cDNAs may really be the yolk BBP cDNAs, which have been reported to be secreted from the liver into the egg [4].

If we try to fit the characteristics of the found BBP candidates to those previously associated with BBPs, BBP-B looks more promising. It has similar low pI [7], its C-terminal extension makes its molecular mass closer to that determined for BBP-II [10] and it is most probably non-glycosylated as is BBP-II [10]. What is then the role of the BBP-A gene? Is it the mysterious BBP isolated from egg-white [6] or some unknown chicken BBP? It is evident that we need to continue the database queries and/or start cloning BBP genes to clarify this puzzle. In addition, we need to produce these found putative BBPs as recombinant proteins to investigate whether their properties are in agreement with the previous findings and the models of the present study. Furthermore, these new proteins can serve as a source for development of new tools for life sciences.

For example, it could be possible to construct a chimeric avidin-BBP-dimer [37] to adjust the ligand-binding properties of the resultant hybrid protein.

## Conclusion

We have identified two putative genes and cDNAs for chicken egg-yolk biotin-binding proteins from NCBI database and chicken genome database. The genomic location and the structures of the found genes and the proteins they encode link clearly BBPs to the avidin family and, moreover, give an insight to the evolutionary history of this gene family. Our molecular modelling results support many preceding observations concerning the biochemical properties of BBPs but also impugn some of the previous hypothesis. Most importantly, the gene/cDNA structures provided no evidences of proteolytic processing of pseudotetramers to tetramers that has been presented as a possible maturation process for BBPs, i.e. conversion of BBP-I to BBP-II.

## Methods

### Database queries and sequence analyses

The N-terminal sequences VEIKXQLSGLWENEQDSLMEISALADDGG and VERKXQLSGLWENEQDSLMEISALADDLEN [4] were used to search the deposited collection of chicken cDNAs [15] by using TBlastn at the NCBI web site. The obtained cDNA sequences were used to find the corresponding genes and their genomic locations by searching the chicken genome database at the Ensembl web site [38] using blastn. Furthermore, cDNA of avidin [39] and *AVRs *[16] were used as search strings from this database. The intron/exon structures of these putative genes were analyzed. DNA sequences of *AVD*, *AVR2*, *AVR4*, *AVR6*, *AVR-A*, *AVR-B*, *AVR-C *and *BBP-A *and *BBP-B *were aligned exon by exon and intron by intron using Clustal X in multiple alignment mode with default values for both pairwise and multiple alignment parameters. Relationships among avidin and other biotin-binding proteins were obtained by the Neighbour Joining method from the p-distance matrix as implemented in Mega software [40]. The Dragon Promoter Finder v. 1.5 [41] was used to predict the promoter regions of the genes. The located promoter regions were further characterised using the transcription factor analysis implemented in the Dragon Promoter Finder program using Match™ [42] with default parameters.

### Structural modelling and polypeptide analysis

The three-dimensional structure of the avidin-biotin complex (PDB code: 2avi [17]) obtained from the Protein Data Bank [43] was used as a template structure in BBP modelling. Sequence alignment of all proteins was made using the MALIGN [44] multiple alignment tool of BODIL [45,46] by using a structure-based sequence comparison matrix [47] with a gap penalty of 40. Comparative models of BBPs were made with Modeller 6v2 [48] according to alignment: disulphide bridges were forced between cysteine residues 3 and 83 in both BBPs and also between cysteine residues 43 and 138 in BBP-B. Furthermore, carboxy-terminal extension of BBP-B (amino acids 129–137) was forced to adopt α-helix conformation. Visual analysis of obtained models was done with the BODIL molecular modelling program. Alignment representation was made using ALSCRIPT [49] and protein representations were made using PyMOL [50]. The putative signal cleavage sites were analyzed by SignalP [21,51]. The theoretical molecular weights, pIs and extinction coefficients were calculated using the program ProtParam [52,53]. The potential N-glycosylation sites and their qualities were studied by NetNglyc [54].

## Abbreviations

AVD, avidin protein; *AVD*, avidin gene; AVR; avidin-related protein; *AVR*, gene coding for avidin like protein; BBP, biotin binding protein; *BBP*, gene coding for biotin-binding protein; CR1, chicken repeat 1; pI, isoelectric point

## Authors' contributions

EAN did molecular modelling analysis. VPH, HRN and OHL did database searches and sequence alignments together with EAN. VPH carried out promoter analysis. AG did genetical analyses of cDNAs and genes. All authors, including MSK, took part in writing of the manuscript.

## References

[B1] Green NM (1975). Avidin. Adv Prot Chem.

[B2] Tuohimaa P, Joensuu T, Isola J, Keinänen R, Kunnas T, Niemelä A, Pekki A, Wallén M, Ylikomi T, Kulomaa M (1989). Development of progestin-specific response in the chicken oviduct. Int J Dev Biol.

[B3] White HB, Whitehead CC (1987). Role of avidin and other biotin-binding proteins in the deposition and distribution of biotin in chicken eggs. Discovery of a new biotin-binding protein. Biochem J.

[B4] Bush L, White HB (1989). Conversion of domains into subunits in the processing of egg yolk biotin-binding protein I. J Biol Chem.

[B5] White HB (1985). Biotin-binding proteins and biotin transport to oocytes. Ann N Y Acad Sci.

[B6] Seshagiri PB, Adiga PR (1987). Identification and molecular characterisation of a biotin-binding protein distinct from avidin of chicken egg white and comparison with yolk biotin-binding protein. Biochim Biophys Acta.

[B7] Meslar HW, Camper SA, White HB (1978). Biotin-binding protein from egg yolk. A protein distinct from egg white avidin. J Biol Chem.

[B8] Wilchek M, Bayer EA (1999). Foreword and introduction to the book (strept)avidin biotin system. Biomol Eng.

[B9] Subramanian N, Adiga PR (1995). Simultaneous purification of biotin-binding proteins-I and -II from chicken egg yolk and their characterization. Biochem J.

[B10] Bush L, McGahan TJ, White HB (1988). Purification and characterization of biotin-binding protein II from chicken oocytes. Biochem J.

[B11] Flower DR, North AC, Sansom CE (2000). The lipocalin protein family: structural and sequence overview. Biochim Biophys Acta.

[B12] Flower DR (2000). Experimentally determined lipocalin structures. Biochim Biophys Acta.

[B13] Chicken genome. http://www.genome.wustl.edu/projects/chicken/.

[B14] Hillier LW, Miller W, Birney E, Warren W, Hardison RC, Ponting CP, Bork P, Burt DW, Groenen MA, Delany ME, Dodgson JB, Chinwalla AT, Cliften PF, Clifton SW, Delehaunty KD, Fronick C, Fulton RS, Graves TA, Kremitzki C, Layman D, Magrini V, McPherson JD, Miner TL, Minx P, Nash WE, Nhan MN, Nelson JO, Oddy LG, Pohl CS, Randall-Maher J, Smith SM, Wallis JW, Yang SP, Romanov MN, Rondelli CM, Paton B, Smith J, Morrice D, Daniels L, Tempest HG, Robertson L, Masabanda JS, Griffin DK, Vignal A, Fillon V, Jacobbson L, Kerje S, Andersson L, Crooijmans RP, Aerts J, van der Poel JJ, Ellegren H, Caldwell RB, Hubbard SJ, Grafham DV, Kierzek AM, McLaren SR, Overton IM, Arakawa H, Beattie KJ, Bezzubov Y, Boardman PE, Bonfield JK, Croning MD, Davies RM, Francis MD, Humphray SJ, Scott CE, Taylor RG, Tickle C, Brown WR, Rogers J, Buerstedde JM, Wilson SA, Stubbs L, Ovcharenko I, Gordon L, Lucas S, Miller MM, Inoko H, Shiina T, Kaufman J, Salomonsen J, Skjoedt K, Wong GK, Wang J, Liu B, Yu J, Yang H, Nefedov M, Koriabine M, Dejong PJ, Goodstadt L, Webber C, Dickens NJ, Letunic I, Suyama M, Torrents D, von Mering C, Zdobnov EM, Makova K, Nekrutenko A, Elnitski L, Eswara P, King DC, Yang S, Tyekucheva S, Radakrishnan A, Harris RS, Chiaromonte F, Taylor J, He J, Rijnkels M, Griffiths-Jones S, Ureta-Vidal A, Hoffman MM, Severin J, Searle SM, Law AS, Speed D, Waddington D, Cheng Z, Tuzun E, Eichler E, Bao Z, Flicek P, Shteynberg DD, Brent MR, Bye JM, Huckle EJ, Chatterji S, Dewey C, Pachter L, Kouranov A, Mourelatos Z, Hatzigeorgiou AG, Paterson AH, Ivarie R, Brandstrom M, Axelsson E, Backstrom N, Berlin S, Webster MT, Pourquie O, Reymond A, Ucla C, Antonarakis SE, Long M, Emerson JJ, Betran E, Dupanloup I, Kaessmann H, Hinrichs AS, Bejerano G, Furey TS, Harte RA, Raney B, Siepel A, Kent WJ, Haussler D, Eyras E, Castelo R, Abril JF, Castellano S, Camara F, Parra G, Guigo R, Bourque G, Tesler G, Pevzner PA, Smit A, Fulton LA, Mardis ER, Wilson RK (2004). Sequence and comparative analysis of the chicken genome provide unique perspectives on vertebrate evolution. Nature.

[B15] Boardman PE, Sanz-Ezquerro J, Overton IM, Burt DW, Bosch E, Fong WT, Tickle C, Brown WR, Wilson SA, Hubbard SJ (2002). A comprehensive collection of chicken cDNAs. Curr Biol.

[B16] Ahlroth MK, Kola EH, Ewald D, Masabanda J, Sazanov A, Fries R, Kulomaa MS (2000). Characterization and chromosomal localization of the chicken avidin gene family. Anim Genet.

[B17] Livnah O, Bayer EA, Wilchek M, Sussman JL (1993). Three-dimensional structures of avidin and the avidin-biotin complex. Proc Natl Acad Sci USA.

[B18] Stumph WE, Kristo P, Tsai MJ, O'Malley BW (1981). A chicken middle-repetitive DNA sequence which shares homology with mammalian ubiquitous repeats. Nucleic Acids Res.

[B19] Wallén MJ, Keinänen RA, Kulomaa MS (1996). Two chicken repeat one (CR1) elements lacking a silencer-like region upstream of the chicken avidin-related genes Avr4 and Avr5. Biochim Biophys Acta.

[B20] Keinänen RA, Wallen MJ, Kristo PA, Laukkanen MO, Toimela TA, Helenius MA, Kulomaa MS (1994). Molecular cloning and nucleotide sequence of chicken avidin-related genes 1–5. Eur J Biochem.

[B21] Nielsen H, Engelbrecht J, Brunak S, von Heijne G (1997). Identification of prokaryotic and eukaryotic signal peptides and prediction of their cleavage sites. Protein Eng.

[B22] Camproux AC, Gautier R, Tuffery P (2004). A hidden markov model derived structural alphabet for proteins. J Mol Biol.

[B23] Freitag S, Le Trong I, Klumb LA, Chu V, Chilkoti A, Stayton PS, Stenkamp RE (1999). X-ray crystallographic studies of streptavidin mutants binding to biotin. Biomol Eng.

[B24] Weber PC, Ohlendorf DH, Wendoloski JJ, Salemme FR (1989). Structural origins of high-affinity biotin binding to streptavidin. Science.

[B25] Cowan SW, Newcomer ME, Jones TA (1990). Crystallographic refinement of human serum retinol binding protein at 2A resolution. Proteins.

[B26] Laitinen OH, Marttila AT, Airenne KJ, Kulik T, Livnah O, Bayer EA, Wilchek M, Kulomaa MS (2001). Biotin induces tetramerization of a recombinant monomeric avidin. A model for protein-protein interactions. J Biol Chem.

[B27] Laitinen OH, Hytönen VP, Ahlroth MK, Pentikäinen OT, Gallagher C, Nordlund HR, Ovod V, Marttila AT, Porkka E, Heino S, Johnson MS, Airenne KJ, Kulomaa MS (2002). Chicken avidin-related proteins show altered biotin-binding and physico-chemical properties as compared with avidin. Biochem J.

[B28] Hytönen VP, Nyholm TK, Pentikäinen OT, Vaarno J, Porkka EJ, Nordlund HR, Johnson MS, Slotte JP, Laitinen OH, Kulomaa MS (2004). Chicken Avidin-related Protein 4/5 Shows Superior Thermal Stability when Compared with Avidin while Retaining High Affinity to Biotin. J Biol Chem.

[B29] Nordlund HR, Hytönen VP, Laitinen OH, Uotila ST, Niskanen EA, Savolainen J, Porkka E, Kulomaa MS (2003). Introduction of histidine residues into avidin subunit interfaces allows pH-dependent regulation of quaternary structure and biotin binding. FEBS Lett.

[B30] Ahlroth MK, Ahlroth P, Kulomaa MS (2001). Copy-number fluctuation by unequal crossing-over in the chicken avidin gene family. Biochem Bioph Res Co.

[B31] Ahlroth MK, Grapputo A, Laitinen OH, Kulomaa MS (2001). Sequence features and evolutionary mechanisms in the chicken avidin gene family. Biochem Bioph Res Co.

[B32] Laitinen OH, Airenne KJ, Marttila AT, Kulik T, Porkka E, Bayer EA, Wilchek M, Kulomaa MS (1999). Mutation of a critical tryptophan to lysine in avidin or streptavidin may explain why sea urchin fibropellin adopts an avidin-like domain. FEBS Lett.

[B33] Sano T, Cantor CR (1995). Intersubunit contacts made by tryphtophan 120 with biotin are essential for both strong biotin binding and biotin-induced tighter subunit association of streptavidin. Proc Natl Acad Sci USA.

[B34] Laitinen OH, Nordlund HR, Hytönen VP, Uotila ST, Marttila AT, Savolainen J, Airenne KJ, Livnah O, Bayer EA, Wilchek M, Kulomaa MS (2003). Rational design of an active avidin monomer. J Biol Chem.

[B35] Pagano A, Giannoni P, Zambotti A, Sanchez D, Ganfornina MD, Gutierrez G, Randazzo N, Cancedda R, Dozin B (2004). Phylogeny and regulation of four lipocalin genes clustered in the chicken genome: evidence of a functional diversification after gene duplication. Gene.

[B36] Sanchez D, Ganfornina MD, Gutierrez G, Marin A (2003). Exon-intron structure and evolution of the Lipocalin gene family. Mol Biol Evol.

[B37] Nordlund HR, Laitinen OH, Hytönen VP, Uotila ST, Porkka E, Kulomaa MS (2004). Construction of a dual chain pseudotetrameric chicken avidin by combining two circularly permuted avidins. J Biol Chem.

[B38] Ensembl Chicken Genome Server. http://www.ensembl.org/Gallus_gallus/.

[B39] Gope ML, Keinanen RA, Kristo PA, Conneely OM, Beattie WG, Zarucki-Schulz T, O'Malley BW, Kulomaa MS (1987). Molecular cloning of the chicken avidin cDNA. Nucleic Acids Res.

[B40] Kumar S, Tamura K, Nei M (2004). MEGA3: Integrated software for Molecular Evolutionary Genetics Analysis and sequence alignment. Brief Bioinform.

[B41] Bajic VB, Seah SH, Chong A, Zhang G, Koh JL, Brusic V (2002). Dragon Promoter Finder: recognition of vertebrate RNA polymerase II promoters. Bioinformatics.

[B42] Kel AE, Gossling E, Reuter I, Cheremushkin E, Kel-Margoulis OV, Wingender E (2003). MATCH: A tool for searching transcription factor binding sites in DNA sequences. Nucleic Acids Res.

[B43] Berman HM, Westbrook J, Feng Z, Gilliland G, Bhat TN, Weissig H, Shindyalov IN, Bourne PE (2000). The Protein Data Bank. Nucleic Acids Res.

[B44] Johnson MS, Overington JP, Blundell TL (1993). Alignment and searching for common protein folds using a data bank of structural templates. J Mol Biol.

[B45] Structural Bioinformatics Laboratory: Bodil.

[B46] Lehtonen JV, Still DJ, Rantanen VV, Ekholm J, Björklund D, Iftikhar Z, Huhtala M, Repo S, Jussila A, Jaakkola J, Pentikainen OT, Nyrönen T, Salminen TA, Gyllenberg M, Johnson M (2004). BODIL: a molecular modeling environment for structure-function analysis and drug design. J Comput Aided Mol Des.

[B47] Johnson MS, May AC, Rodionov MA, Overington JP (1996). Discrimination of common protein folds: application of protein structure to sequence/structure comparisons. Method Enzymol.

[B48] Sali A, Blundell TL (1993). Comparative protein modelling by satisfaction of spatial restraints. J Mol Biol.

[B49] Barton GJ (1993). ALSCRIPT: a tool to format multiple sequence alignments. Protein Eng.

[B50] DeLano WL (2002). The PyMOL Molecular Graphics System.

[B51] SignalP 3.0 Server. http://www.cbs.dtu.dk/services/SignalP/.

[B52] ExPASy-ProtParam tool. http://au.expasy.org/tools/protparam.html.

[B53] Gill SC, von Hippel PH (1989). Calculation of protein extinction coefficients from amino acid sequence data. Anal Biochem.

[B54] NetNGlyc 1.0 Server. http://www.cbs.dtu.dk/services/NetNGlyc/.

[B55] Ahlroth MK, Kola EH, Ewald D, Masabanda J, Sazanov A, Fries R, Kulomaa MS (2000). Characterization and chromosomal localization of the chicken avidin gene family. Anim Genet.

[B56] Kumar S, Tamura K, Nei M (2004). MEGA3: Integrated software for Molecular Evolutionary Genetics Analysis and sequence alignment. Brief Bioinform.

[B57] Laitinen OH, Hytönen VP, Ahlroth MK, Pentikäinen OT, Gallagher C, Nordlund HR, Ovod V, Marttila AT, Porkka E, Heino S, Johnson MS, Airenne KJ, Kulomaa MS (2002). Chicken avidin-related proteins show altered biotin-binding and physico-chemical properties as compared with avidin. Biochem J.

[B58] Livnah O, Bayer EA, Wilchek M, Sussman JL (1993). Three-dimensional structures of avidin and the avidin-biotin complex. Proc Natl Acad Sci USA.

